# Carcinoma-Associated Fibroblasts Lead the Invasion of Salivary Gland Adenoid Cystic Carcinoma Cells by Creating an Invasive Track

**DOI:** 10.1371/journal.pone.0150247

**Published:** 2016-03-08

**Authors:** Jiao Li, Zhuqiang Jia, Jing Kong, Fuyin Zhang, Shimeng Fang, Xiaojie Li, Wuwei Li, Xuesong Yang, Yong Luo, Bingcheng Lin, Tingjiao Liu

**Affiliations:** 1 College of Stomatology, Dalian Medical University, Dalian, China; 2 Department of Oral Surgery, the First Affiliated Hospital, Dalian Medical University, Dalian, China; 3 Department of Oral Surgery, the Second Affiliated Hospital, Dalian Medical University, Dalian, China; 4 Department of Biochemistry and Molecular Biology, Dalian Medical University, Dalian, China; 5 Faculty of Chemical, Environmental and Biological Science and Technology, Dalian Technology University, Dalian, China; 6 Department of Biotechnology, Dalian Institute of Chemical Physics, Chinese Academy of Sciences, Dalian, China; University of California, San Diego, UNITED STATES

## Abstract

Carcinoma-associated fibroblasts (CAFs) are critical in determining tumor invasion and metastasis. However the role of CAFs in the invasion of salivary gland adenoid cystic carcinoma (ACC) is poorly understood. In this study, we isolated primary CAFs from two ACC patients. ACC-derived CAFs expressed typical CAF biomarkers and showed increased migration and invasion activity. Conditioned medium collected from CAFs significantly promoted ACC cell migration and invasion. Co-culture of CAFs with ACC cells in a microfluidic device further revealed that CAFs localized at the invasion front and ACC cells followed the track behind the CAFs. Interfering of both matrix metalloproteinase and CXCL12/CXCR4 pathway inhibited ACC invasion promoted by CAFs. Overall, our study demonstrates that ACC-derived CAFs exhibit the most important defining feature of CAFs by promoting cancer invasion. In addition to secretion of soluble factors, CAFs also lead ACC invasion by creating an invasive track in the ECM.

## Introduction

Adenoid cystic carcinoma (ACC) is one of the most frequent malignant tumors arising in the salivary glands [1], representing about 2–6.5% of tumors of the head and neck region[2]. It shows a strong capacity for local invasion and a high incidence of distant metastasis [3]. ACC cells can be found at a considerable distance beyond the clinical boundaries of the tumor. In addition, tumor cells can extensively invade bone before there is radiographic evidence of osseous destruction. Elucidating the mechanisms of ACC invasion might thus provide new strategy for ACC therapy.

Genetic and cell biology studies indicate that tumor progression is not just determined by malignant cancer cells themselves, but also by tumor stroma[4]. Evidence is increasing that the fibroblasts in tumor stroma remain permanently activated and serve as important promoters of tumor growth, invasion and metastasis[5–8]. These activated fibroblasts are often termed as cancer-associated fibroblasts (CAFs), tumor-associated fibroblasts, or myofibroblasts due to their expression of α-smooth muscle actin (α-SMA)[5, 9]. However, α-SMA expression alone will not identify all CAFs[10, 11]. Other CAF biomarkers in use include vimentin (VIM), fibroblast activation protein (FAP), and fibroblast-specific protein 1 (FSP-1)[4, 11].In addition, cytokeratin (CK) is negative in CAFs which differentiates them from epithelium-originated cancer cells [12]. CAFs produce a variety of cytokines and chemokines that are involved in cross-talk between the CAFs and the tumor and other stromal cells to affect tumor growth and metastasis[13]. These factors can promote tumor cell proliferation and invasion, stimulate angiogenesis, and recruit bone marrow-derived cells or immune cells into the growing tumor[5, 14, 15]. In addition, CAFs are a source of matrix metalloproteinases (MMPs) as extracellular matrix (ECM)-degrading proteases. In breast cancer, CAFs promote cancer cell growth, angiogenesis, and invasion by C-X-C motif chemokine 12 (CXCL12), MMP9, and MMP14 [9]. In prostate cancer, CAFs have been shown to affect the proliferation and facilitate the invasiveness of cancer cells by CXCL12, CXCL14, MMP2 and MMP3[16, 17]. In lung cancer, CAFs promote the proliferation and invasiveness of cancer cells by high expression of Forkhead box F1 and CXCL12[18, 19].CAFs from squamous cell carcinoma of the head, neck and esophagus secrete hepatocyte growth factor to promote cancer invasion[20]. Our previous study demonstrated that ACC-derived CAFs promoted ACC cell invasion in a 3D matrix in a spheroid fashion[21]. Furthermore, we demonstrated that ACC-derived CAFs showed high levels of MMP2 and CXCL12 expression, which might be related to the aggressive growth behavior of ACC[22].

The purpose of this study was to further investigate the biological characteristics of ACC-derived CAFs and the effects of CAFs on ACC invasion. We found that ACC-derived CAFs showed a strong capacity for migration and invasion. CAFs promoted ACC invasion by creating an invasive track by a method other than their secretion of soluble factors.

## Materials and Methods

All studies involving human materials were approved by the Research Ethics Committee, Dalian Medical University, China. The work described was carried out in accordance with The Code of Ethics of the World Medical Association (Declaration of Helsinki) for experiments involving humans. Written consents were obtained from the participants for experimentation with human subjects.

### 2.1. Primary cell isolation, general cell culture and immunofluorescent staining

Two cases of ACCs were obtained from The First and Second Affiliated Hospital of Dalian Medical University. Diagnosis was made using histological sections stained with hematoxylin and eosin (HE). Immunohistochemical staining, primary cell isolation and characterization were performed as described previously [22]. The primary fibroblasts isolated from two ACC patients were named “CAF-A1” and “CAF-A2”. The fibroblasts isolated from normal gingival tissues were named “NF”. They were culture in Dulbecco’s Modified Eagle Medium: Nutrient Mixture F-12 (DMEM/F12; Hyclone, Logan, UT, USA) with 10% fetal bovine serum (FBS; ScienCell, Carlsbad, CA, USA), 100U/mL penicillin and 100U/mL streptomycin (Hyclone). We used cells passaged up to five population doublings for initial characterization by immunofluorescence staining. Primary antibodies targeting pan-CK, VIM, α-SMA, FAP and FSP-1 were used (Table 1).

**Table 1:** Clone, dilution and sources of primary antibodies.

Antibody	Clone	Dilution	Source
Pan-CK	AK1/AE3	1:100	ZSGB-BIO
VIM	V9	1:200	Invitrogen
α-SMA	1A4	1:100	Zeta Corporation
FAP	Polyclone	1:100	Abcam
FSP-1	Polyclone	1:100	Abcam

SACC-LM and SACC-83 cell lines were kind gifts from Prof. Jing Xiao [23]. They were cultured in DMEM/F12 supplemented with 10% FBS with100 U/mL penicillin and100 U/mL streptomycin at 37°Cwith 5% CO_2_ and 95% relative humidity.

### 2.2. Preparation of conditioned medium

CAF-A1, CAF-A2 and NF were cultured in complete growth DMEM/F12 to approximately 80% confluence, when the medium was changed to FBS-free DMEM/F12. After a further 48h, the cell culture medium was collected and centrifuged at 1200×*g* for 10min. The supernatant was collected and stored at –80°C as conditioned medium (CM).

### 2.3. Wound healing assay

The lateral motility of CAF-A1, CAF-A1-2 and NF cells was examined by a wound healing assay. The cells (5× 10^5^ cells per well) were seeded into six-well plates. After 24h, three wounds were created in each well using a P1000 pipette tip. The cells were then rinsed once with PBS and cultured for another48 or 72 h. Motility was expressed as the cell migration rate. The wound area at the start and end of each experiment was recorded using an inverted microscope (Olympus IX 71) and calculated using Image-Pro Plus 6.0. The cell migration rate was determined as the decrease in the wound area normalized to the wound area at the initial time-point.

### 2.4. Cell migration and invasion assay using Transwell® plates

Cell migration and invasion abilities were assessed using Transwell® plates (Corning, Corning, NY, USA). For the cell migration assay, 2×10^4^ cells in 200 μL serum-free medium were seeded directly into the wells of Transwell® chambers with 8 μm-pore membranes. The cell invasion assay was performed using Transwell® chambers coated with Matrigel™ (Corning), with medium containing20% FBS, or CM from different fibroblasts, separately added into the lower chamber. After incubating for 48h at 37°Cin5%CO_2_, the medium in the chambers was removed, the cells were fixed in 4% paraformaldehyde for 20min, washed three times with PBS, and stained with 1% methylrosa (Solarbio, Beijing, China) for 20min. The cells adhering to the upper surface of the membrane were removed using a cotton applicator. The cells on the lower side of the membrane were counted. Five random high-power fields (× 200) were selected and the mean number of cells was identified as the cell migration or invasion number. The data represent at least three experiments performed in triplicate (mean ± standard error).

### 2.5. Cell invasion assay using a microfluidic model

To evaluate the invasive potential of CAFs, a microfluidic device developed in our previous study was used[24]. Matrigel™ (Corning), as the substitute for ECM, was loaded into the matrix channel. To assess the invasive activity of fibroblasts, CAF-A1, CAF-A2 and NF were seeded into the cell culture channel in serum-free medium and medium with 20% FBS was loaded into the stimulation channel. After 48 h, actin filaments and nuclei were stained with rhodamine-phalloidin (Invitrogen, Carlsbad, CA, USA) and DAPI, respectively. To assess the effect of fibroblasts on ACC invasion, fibroblasts and ACC cells were labeled with Celltracker Red CMTPX (Invitrogen) and PKH67 green fluorescent cell linker (Sigma) respectively. Then the fibroblast-ACC mixture was seeded into the cell culture channel and cultured in serum-free medium. Medium containing 20% FBS was loaded into the stimulation channel and the device was placed inside a 37°C incubator with 5% CO_2_ for 48 h. MMP inhibition experiments were carried out by using GM 6001 (Millipore, MA, USA), which was added to the cell culture medium at the concentration of 100 mg/mL. The CXCL12 inhibition experiment was conducted by adding AMD3100 (Sigma) to the cell culture medium. The invasion of cells into the Matrigel™ was recorded with an inverted microscope (Olympus IX 71). Cell invasion area was determined as the area invaded by cells in the matrix channel and measured by Image-Pro Plus 6.0. The data represent at least three experiments performed in triplicate (mean ± standard error).

### 2.6. Statistical analysis

Statistical analysis was performed using SPSS version 13.0 for Windows. The differences in expression of target proteins between ACCs and normal salivary glands were analyzed by Student’s *t* test. Statistical significance was defined as *P*< 0.05.

## Results

### 3.1. CAFs expressed typical biomarkers of activated fibroblasts

**Fig 1:**
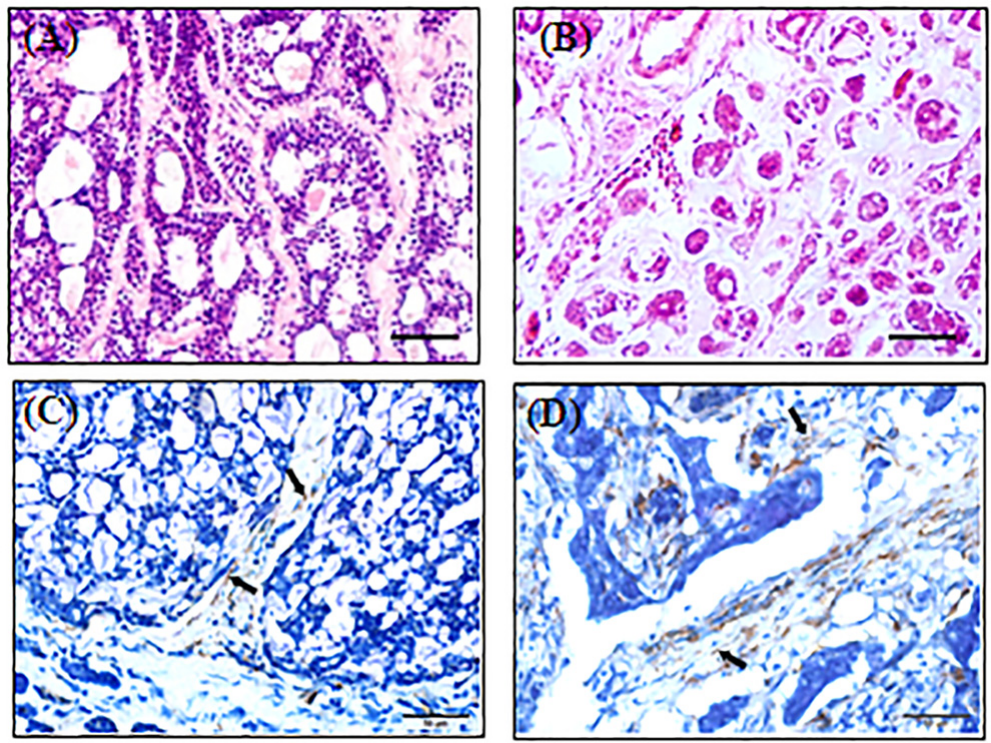
HE staining showing the histopathological features of two ACCs. (A) Case 1. Tumor cells arranged in typical cribriform growth patterns. (B) Case 2. Tumor cells arranged in tubular growth patterns. (C) Immunohistochemical staining of α-SMA for Case1. Arrows pointed to the CAFs in the stroma of case 1. (D) Immunohistochemical staining of α-SMA for Case2. Arrows pointed to the CAFs in the stroma of case 2. Scale bar = 100μm

Typical histopathological appearances of ACC were recognized in the two cases (Fig 1A and 1B). Tumor cells arranged in cribriform or tubular growth patterns in the stroma. Numerous α-SMA-positive cells were observed in the stroma by immunohistochemical staining (Fig 1C and 1D), suggesting the existence of CAFs in the two ACC cases.

**Fig 2:**
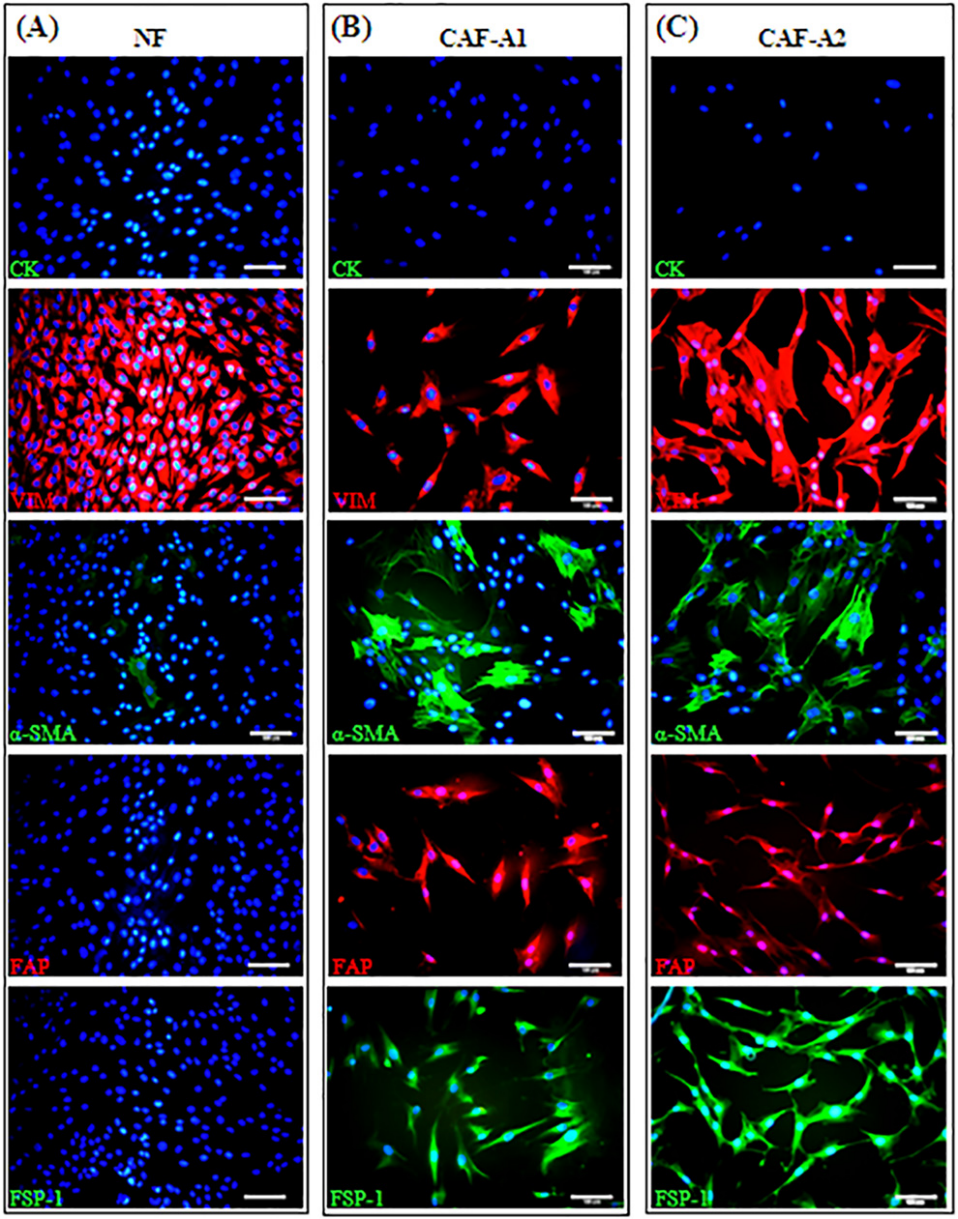
Immunofluorescent staining for pan-CK, VIM, α-SMA, FAP, FSP-1 in NF, CAF-A1, and CAF-A2. (A) NF was negative for pan-CK, α-SMA, FAP, FSP-1 and positive for VIM. (B) CAF-A1 was negative for pan-CK and positive for VIM, α-SMA, FAP, FSP-1.(C) CAF-A2was negative for pan-CK and positive for VIM, α-SMA, FAP, FSP-1. Scale bar = 100 μm

CAF-A1 and CAF-A2 were successfully isolated from the two ACC cases. NF was isolated from normal gingival tissues. All three cell types exhibited typical fibroblastic morphology with cytoplasmic processes on a 2D surface. They were negative for pan-CK and positive for VIM (Fig 2A–2C).NF were negative for α-SMA, FAP, and FSP-1 (Fig 2A). By contrast, a great number of CAF-A1 and CAF-A2 were positive for α-SMA, and almost all CAF-A1 and CAF-A2 were positive for FAP and FSP-1 (Fig 2B and 2C).

### 3.2. CAFs showed increased migration and invasion ability

**Fig 3:**
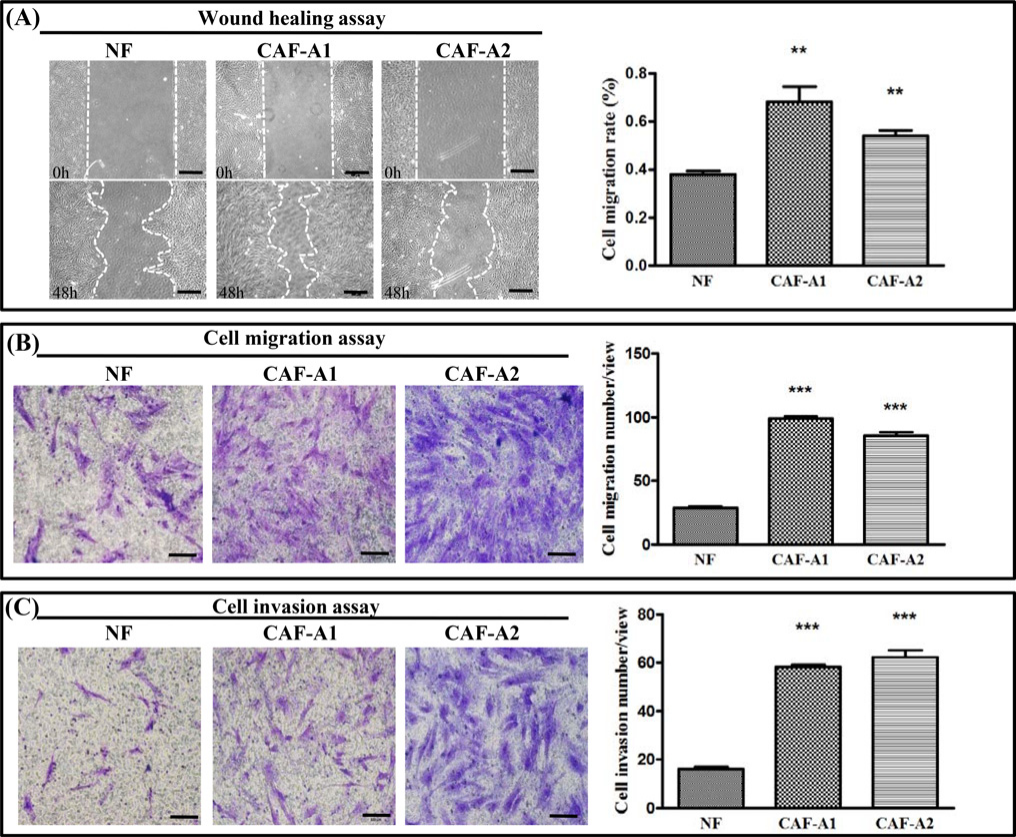
Migration and invasion of NF, CAF-A1, and CAF-A2. (A) Wound healing assay. Both CAF-A1 and CAF-A2 showed significantly increased migration activity compared to NF. (B) Transwell® migration assay. Significantly more CAF-A1 and CAF-A2 cells transmigrated through the pores of the Transwell® than NF. (C) Transwell® invasion assay. Significantly more CAF-A1 and CAF-A2 cells invaded through the matrix coating on the Transwell® membrane compared toNF. (n = 3)

The migration abilities of CAF-A1 and CAF-A2 were assessed. The results showed that CAF-A1 and CAF-2 both exhibited significantly higher migration ability than NF, as assessed by both the wound healing assay and Transwell® assay (Fig 3A and 3B). To evaluate the invasion abilities of CAF-A1 and CAF-A2, we first performed the Transwell® assay with a Matrigel™ coating. This revealed that both CAF-A1 and CAF-A2 had significantly higher invasion ability than NF (Fig 3C).

**Fig 4:**
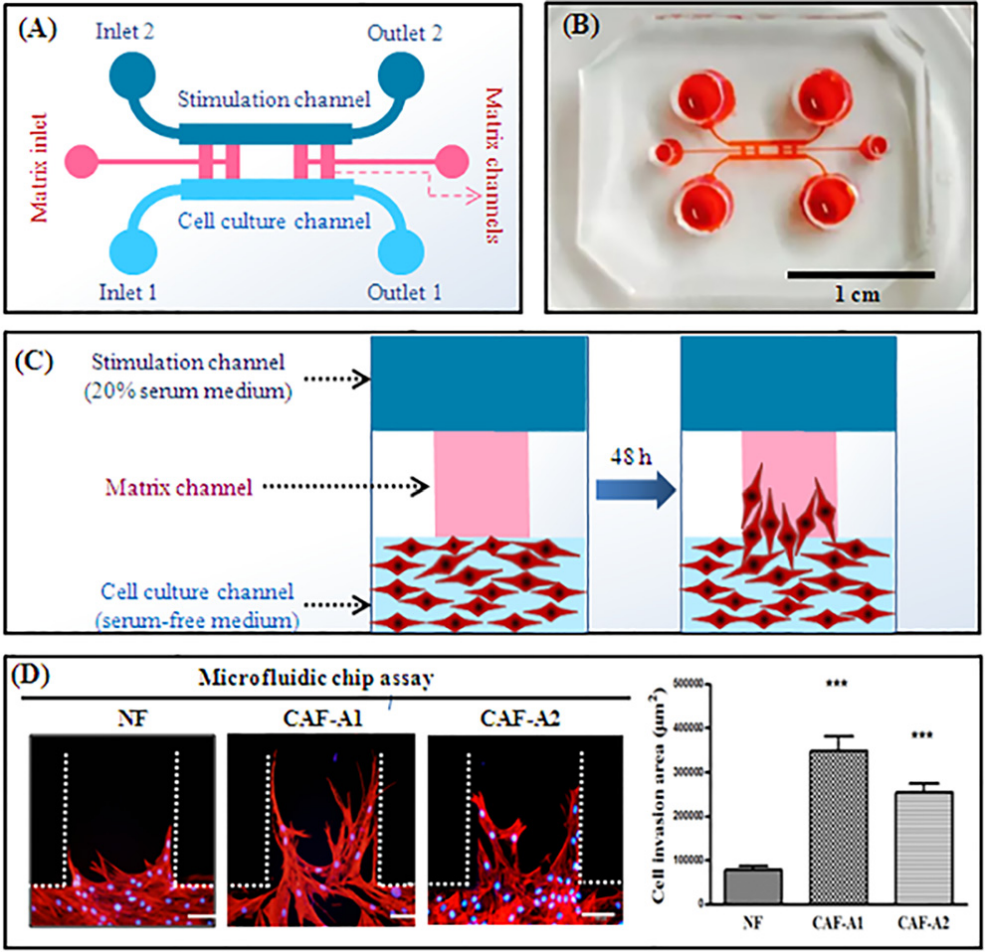
Microfluidic-based cell invasion assay. (A) Illustration of the microfluidic device. (B) Photo of the microfluidic device filled with red stain. (C) Experimental design. Fibroblasts (red) were cultured in the cell culture channel of a microfluidic device.(D) Cell invasion assay using a microfluidic device. The invasion areas of CAF-A1 and CAF-A2 were significantly greater than that of NF. ** *P*< 0.01, *** *P*< 0.001, n = 5. Scale bar = 100 μm

As the Transwell® assay could only assess the vertical invasion of cells, a microfluidic device developed in our previous study was used to investigate the lateral invasion of CAFs and view the invasion process(Fig 4A and 4B).The device is composed of a cell culture channel (light blue), a stimulation channel (dark blue) and four matrix channels (pink). The cell culture channel and stimulation channel have their own inlets and outlets. The matrix channels are located between the cell culture and stimulation channels and have their own inlets. The device is made of PDMS which is translucent. The device is sterilized and bonded to a 6 cm plate before use. The experimental design was showed in Fig 4C. First, Matrigel™ was loaded into the matrix channels. Then NF, CAF-A1, or CAF-A2 in serum-free medium were seeded into the cell culture channel. Medium containing 20% serum was loaded into the stimulation channel. It was found that both CAF-A1 and CAF-A2 invaded into the matrix with long cellular processes (Fig 4D). The invasion areas of CAF-A1 and CAF-A2 were significantly higher than that of NF. These results suggest that both CAF-A1 and CAF-A2 showed increased migration and invasion abilities. No significant differences in migration and invasion abilities between CAF-A1 and CAF-A2 were found.

### 3.3. CAF-derived CM promoted the migration and invasion of ACC cells

**Fig 5:**
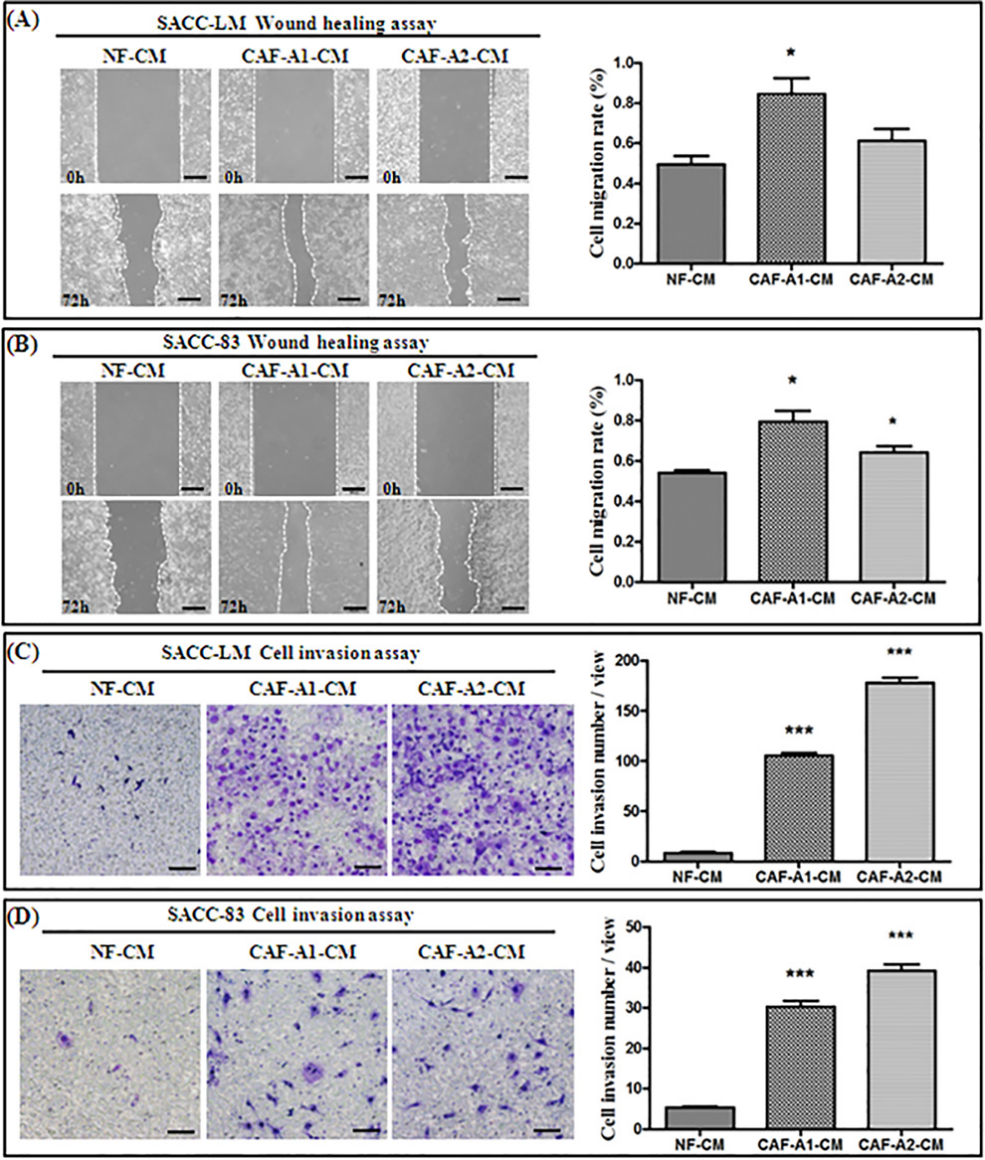
The effects of CM prepared from NF, CAF-A1, and CAF-A2 on the migration and invasion of SACC-LM and SACC-83 cells. (A-B) ACC migration assay. CAF-A1-CM promoted SACC-LM migration significantly more than NF-CM. CAF-A2-CM also promoted SACC-LM migration more than NF-CM, but the difference was not significant (A). Both CAF-A1-CM and CAF-A2-CM promoted SACC-83 migration significantly more than NF-CM (B). (C-D) ACC invasion assay using Transwell® plates. CAF-A1-CM and CAF-A2-CM promoted SACC-LM (C) and SACC-83 (D) invasion significantly more than NF-CM. * *P*< 0.05, *** *P*< 0.001, n = 3. Scale bar = 100 μm

As it has been reported that CAFs secrete various factors which influence cancer cell motility, we assessed the effects of CAF-derived CM on the motility of SACC-LM and SACC-83 cells. The wound healing assay revealed that both CAF-A1- and CAF-A2-derived CM greatly promoted the migration of SACC-LM and SACC-83 cells, compared to NF-CM (Fig 5A and 5B). In particular, CAF-A1-CM significantly increased the migration abilities of both SACC-LM and SACC-83 cells (*P*< 0.05). The Transwell® invasion assay demonstrated that the invasive abilities of both SACC-LM and SACC-83 were increased significantly by both CAF-A1-CM and CAF-A2-CM, compared to NF-CM (*P*< 0.001; Fig 5C and 5D). In the Transwell® assay we observed increased numbers of invaded SACC-LM cells compared to SACC-83cells following induction with CAF-A1- and CAF-A2-CM. This result might be connected to the fact that SACC-LM is a highly metastatic cell line. Overall our results suggest that CAF-A1 and CAF-A2 secrete certain factors in CM that greatly stimulate ACC cell invasion.

### 3.4. CAFs led the invasion of ACC cells

**Fig 6:**
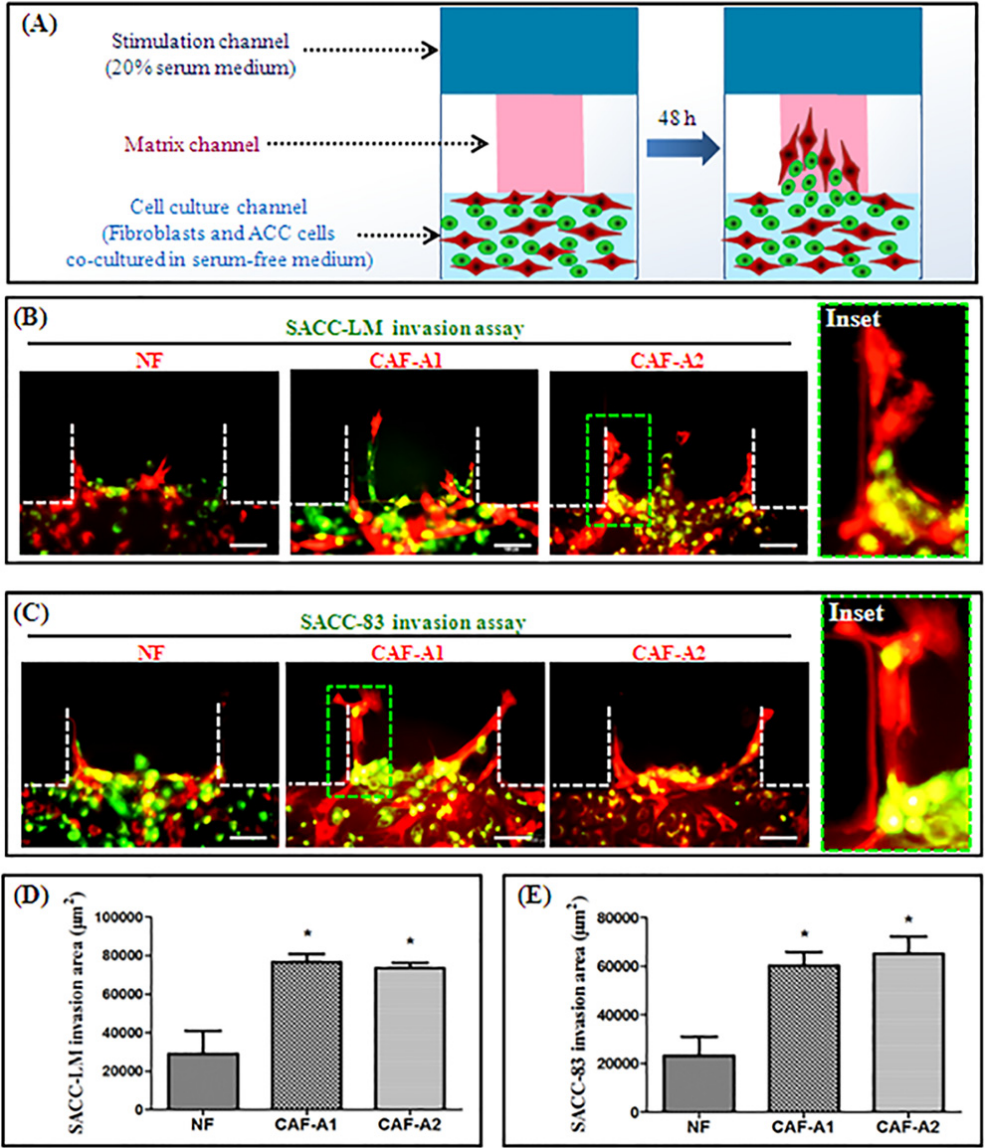
The effects of NF, CAF-A1, and CAF-A2 on the invasion of SACC-LM and SACC-83 cells. (A) Experimental design. Fibroblasts (red) and ACC cells (green) were co-cultured in the cell culture channel of a microfluidic device.(B-C) Co-culture of NF, CAF-A1, or CAF-A2 with ACC cells in a microfluidic device. CAF-A1 and CAF-A2 promoted SACC-LM (B) and SACC-83 (C) invasion. CAFs localized at the invasion front and ACC cells followed the CAFs (Inset). Inset represents the green dotted square. (D-E) The invasion areas of SACC-LM (D) and SACC-83(E) were significantly increased in CAF-A1 and CAF-A2 co-culture groups compared to that in the NF co-culture group. * *P*< 0.05, n = 3. Scale bar = 100 μm

To investigate whether CAFs can promote ACC cell invasion in co-culture, SACC-LM and SACC-83 cells were co-cultured with NF, CAF-A1 or CAF-A2 in the microfluidic model. The experimental design was showed in Fig 6A. First, Matrigel™ was loaded into the matrix channels. Then NF, CAF-A1, or CAF-A2 (red) were seeded into the cell culture channel together with ACC cells (green) and cultured in serum-free medium. Medium containing 20% serum was loaded into the stimulation channel. After co-culture for 48 h, both SACC-LM and SACC-83 cells invaded the matrix together with NF, CAF-A1, and CAF-A2 (Fig 6B and 6C).Close examination revealed that CAF-A1 and CAF-A2 always localized to the invasion front while SACC-LM and SACC-83 followed the track of CAFs into the Matrigel™ behind the CAFs (Fig 6B and 6C insets). By contrast, some SACC-LM or SACC-83 cells were localized to the invasion front in some areas in the NF co-culture groups. Quantification of the results demonstrated that the invasive areas of SACC-LM and SACC-83 in CAF-A1 and CAF-A2 co-culture groups were significantly higher than that in the NF co-culture group (*P*< 0.05; Fig 6D and 6E).

### 3.5. Inhibition of ACC invasion promoted by CAFs

**Fig 7:**
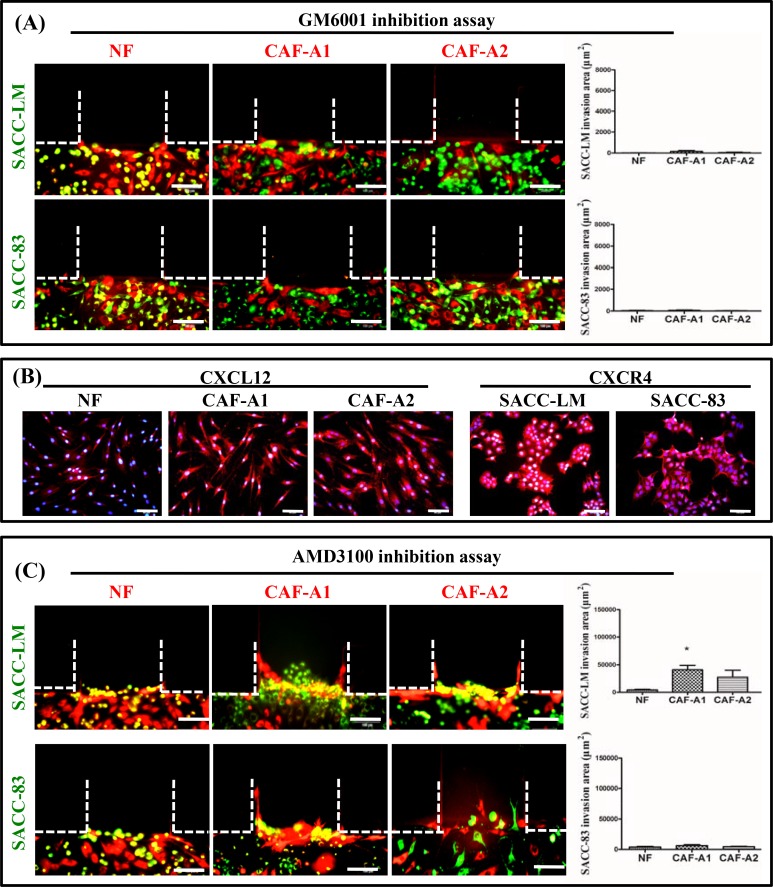
Inhibition of ACC invasion promoted by CAFs. (A) GM6001 inhibited both SACC-LM and SACC-83 invasion promoted by CAF-A1 and CAF-A2. (B) CXCL12 expression was confirmed in CAF-A1 and CAF-A2. NF showed low CXCL12 expression. (C) AMD3100 inhibited SACC-LM and SACC-83 invasion led by CAFs. * *P*< 0.05, n = 3. Scale bar = 100 μm

As MMPs are essential for the remodeling of ECM, MMP inhibition experiments were further carried out using GM6001 to confirm ECM remodeling during CAFs and ACC invasion. It was found that both invasions of CAFs and ACC were inhibited by GM6001 completely (Fig 7A).

CXCL12 expression was confirmed in both CAF-A1 and CAF-A2, while CXCR4 expressed in SACC-LM and SACC-83 cells (Fig 7B). To investigate whether CXCL12/CXCR4 signaling pathway plays a certain role in ACC invasion led by CAFs, AMD3100was utilized in this study. It was found that both SACC-LM and SACC-83 invasion were inhibited by AMD3100 (Fig 7C). Especially, both SACC-LM and SACC-83 did not follow the track of CAFs. Some SACC-LM cells even invaded ahead of CAFs due to their high invasion capability.

## Discussion

ACC tend to invade early and extensively *in vivo*, resulting in poor patient survival and prognosis[2]. Elucidating the mechanism of ACC invasion might therefore provide a novel therapeutic target. Our previous study demonstrated that ACC-derived CAFs might promote cancer invasion by expressing MMP2 and CXCL12 *in vivo*, but it remained unclear how CAFs promote ACC cell invasion.

CAFs have been reported to be important promoters of tumor growth and progression[4], and have been found in the stroma of various cancers. This cell-type is mostly defined based on morphological characteristics and the expression of markers such as VIM, α-SMA, FAP, and FSP-1.VIM is a common marker for mesenchymal cells expressed in both normal fibroblasts and CAFs[12]. Although α-SMA is a biomarker which is widely used to detect CAFs, the functional significance of α-SMA-expressing fibroblasts in tumor stroma is uncertain. Some researchers reported that these α-SMA-expressing fibroblasts promoted tumor growth and angiogenesis[5]. By contrast, transgenic mice with the ability to delete α-SMA-expressing fibroblasts in pancreatic cancer developed invasive, undifferentiated tumors with enhanced hypoxia, epithelial-to-mesenchymal transition, and cancer stem cells, with diminished animal survival[25]. FAP is not expressed by mature tissues except for activated fibroblasts during wound healing and in tumor stroma [4]. It has emerged as a promising candidate for specifically targeting CAFs. Despite its name, FSP-1 is expressed by a variety of cell types within the tumor stroma, including CAFs, macrophages and malignant cells[26]. In addition, FSP-1-expressing fibroblasts in tumor stroma did not express α-SMA to a large extent, indicating the complicated features of CAFs. Thus, it is essential to use several biomarkers along with cell morphology analysis to verify the purity of CAFs. In this study, fibroblasts extracted from two ACC cases were not only positive for common fibroblastic markers such as VIM, but also positive for activated fibroblastic markers such as α-SMA, FAP and FSP-1. In addition, we found that almost all ACC-derived CAFs expressed FAP, while only some CAFs expressed α-SMA. These findings suggest the cellular heterogeneity of ACC-derived CAFs.

The most important defining feature of CAFs compared to normal fibroblasts is their capacity to stimulate cancer invasion and metastasis. CAFs are the main source of ECM in tumor stroma and can promote cancer invasion by remodeling the ECM. In breast cancer, CAFs produce linear bundles of ECM and this radial alignment of type I collagen fibers facilitates local invasion and correlates with poor disease-free survival[27]. Lysyl oxidase (LOX) and LOX-like protein 2 (LOXL2) produced by CAFs promote cross-linking of fibrillar type I collagen and result in matrix stiffening and consequent mechanotactic cues that support cancer cell invasion[28]. CAF-promoted invasion can also be achieved by inducing epithelial–mesenchymal transition (EMT). CAFs have been shown to induce EMT and ultimately metastasis of murine prostate cancer cells by secreting CXCL12[29]. Platelet-derived growth factor (PDGF) receptor signaling is a major functional determinant of CAFs. PDGF-stimulated fibroblasts secrete stanniocalcin-1 and stimulate invasion of colorectal cancer cells through upregulation of VIM[30]. CAFs can also promote invasion by inducing expression of MMPs by cancer cells or by expressing MMPs themselves. Oral squamous cell carcinoma (OSCC)-derived CAFs secrete CCL7 to up-regulate MMP2 expression by OSCC cells and thus increase cancer invasion [31]. Our previous study revealed that ACC-derived CAFs express high levels of MMP2 *in vivo* and *in vitro*. In the present study, we demonstrate that CM prepared from two ACC-derived CAFs promotes the migration and invasion of ACC cells. Compared to the effect of CAFs on ACC cell migration, CAFs significantly promoted ACC cell invasion (*P*< 0.001). In particular, SACC-83 is a low invasive ACC cell line and seldom invades matrix without CAF stimulation. Therefore, we hypothesize that CAFs secrete certain soluble factors that stimulate ACC cell invasion.

It has been reported that the CAF network may serve as a guiding structure to direct invasion. Individual CAFs create tracks in the type I collagen-rich mesh-work through both proteolytic and force-mediated modifications. Imaging of collectively invading co-cultures of carcinoma cells and stromal fibroblasts reveals that the leading cell is always a fibroblast and that carcinoma cells move within tracks in the ECM behind the fibroblasts[32]. To investigate the mechanisms of CAF-promoted ACC cell invasion apart from their secretion of certain factors, we co-cultured CAFs and ACC cells on a microfluidic device. Microfluidic technology has been emerging as an ideal platform in the biological and medical sciences, requiring less time, reduced sample consumption and low cost. It creates new opportunities for spatial and temporal control of cell growth and stimuli. In addition, cell behavior can be observed in real time because polydimethylsiloxane (PDMS), the material used to create a microfluidic device, is translucent and oxygen permeable[33]. Using our microfluidic device, we found that CAFs are always located in the invasion front while ACC cells follow the track created by CAFs. By contrast, some ACC cells were located at the invasion front in the NF co-culture group. Combining these results with the data of higher invasion ability of CAFs compared to NF, we hypothesize that CAFs degrade the ECM and create an invasion track to lead ACC invasion. MMP inhibitor restrained both CAF and ACC invasion, suggesting ECM degrading is essential for cancer invasion. Furthermore, we demonstrated that CAFs expressed CXCL12 and interfering CXCL12/CXCR4 pathway by AMD3100 inhibited ACC invasion led by CAFs.

In conclusion, we demonstrate that ACC-derived CAFs not only express typical CAF biomarkers, but also present the most important defining feature of CAFs by promoting cancer invasion. In addition to secretion of soluble factors, CAFs lead ACC invasion by creating an invasive track in the ECM.
